# Characteristics and Treatment Rate of Patients With Hepatitis C Virus Infection in the Direct-Acting Antiviral Era and During the COVID-19 Pandemic in the United States

**DOI:** 10.1001/jamanetworkopen.2022.45424

**Published:** 2022-12-07

**Authors:** Vy H. Nguyen, Leslie Kam, Yee Hui Yeo, Daniel Q. Huang, Linda Henry, Ramsey Cheung, Mindie H. Nguyen

**Affiliations:** 1 Division of Gastroenterology and Hepatology, Stanford University Medical Center, Palo Alto, California; 2 Division of General Internal Medicine, Cedars-Sinai Medical Center, Los Angeles, California; 3 Division of Gastroenterology and Hepatology, Department of Medicine, National University Hospital, Singapore; 4 Department of Medicine, Yong Loo Lin School of Medicine, National University of Singapore, Singapore; 5 Division of Gastroenterology and Hepatology, Veterans Affairs Palo Alto Health Care System, Palo Alto, California; 6 Department of Epidemiology and Population Health, Stanford University School of Medicine, Palo Alto, California

## Abstract

**Importance:**

Clinical data on hepatitis C virus (HCV) treatment rates in the United States are sparse.

**Objective:**

To evaluate HCV treatment rates in the era of direct-acting antivirals (DAAs).

**Design, Setting, and Participants:**

This retrospective cohort study used data from the deidentified Optum Cliniformatics Data Mart Database (2014-2021) on patients with HCV in the DAA and COVID-19 eras. The database includes patients with private health insurance in the US.

**Main Outcomes and Measures:**

The treatment rate and changes over time were assessed with adjusted log-binomial regression, and factors associated with treatment were examined using multivariable logistic regression.

**Results:**

A total of 133 348 patients with HCV (79 567 [59.7%] men; mean [SD] age, 59.7 [12.3] years; 4448 [3.3%] Asian, 24 662 [18.5%] Black, and 74 750 [56.1%] White individuals) were included; 38 180 (26.8%) had HCV RNA data, and of those, 20 277 (53.1%) had positive HCV RNA. Overall, 13 214 patients with positive HCV RNA tests (65.2%) received DAA treatment; 6456 of 6634 patients treated with DAAs (97.3%) achieved sustained virologic response. After adjusting for age, sex, and race and ethnicity, the treatment rate in 2018 was 0.5 times greater than the rate in 2014 (adjusted prevalence ratio, 1.50; 95% CI, 1.42-1.59) but declined after 2018, decreasing from 64.8% to 61.2%, and especially after 2019, when it decreased to less than 60% (*P* < .001). The number of patients with viremic HCV identified in between April 2020 and March 2021 also decreased to 496 from 2761 and 3258 in the preceding 2 years. Receiving care from a gastroenterologist or infectious disease specialist with advanced care practitioner (ie, nurse practitioner, physician assistant, or clinical nurse specialist) was independently associated with greater odds of DAA treatment (adjusted odds ratio [aOR], 1.64; 95% CI, 1.07-1.50). Patients with decompensated cirrhosis and/or hepatocellular carcinoma (HCC) were 31% less likely to receive treatment compared with those without (aOR, 0.69; 95% CI, 0.54-0.90).

**Conclusions and Relevance:**

In this cohort study, less than two-thirds of insured patients with viremic HCV received DAA treatment, with declines in both the treatment rate and the number of viremic HCV diagnoses since 2019 and especially during the COVID-19 pandemic. Further efforts are needed to increase HCV diagnosis and treatment, especially for those with cirrhosis and HCC. An urgent call for nationwide actions to improve access to DAA treatment, community outreach programs, and specialists through referral pipelines is needed in the United States to stay on track to meet the World Health Organization goal of reducing the burden of viral hepatitis with the eventual goal to eliminate viral hepatitis.

## Key Points

**Question:**

How has the treatment rate for hepatitis C virus infection with direct-acting antivirals (DAAs) changed over time?

**Findings:**

In this cohort study of 20 277 patients with viremic hepatitis C and private health insurance in the United States, as of 2020, there was a suboptimal DAA treatment rate of 65.2% and a declining treatment rate beginning in 2019. Patients with hepatocellular carcinoma and decompensated cirrhosis were 30% less likely to receive DAA treatment than those without either condition.

**Meaning:**

These findings suggest that DAA treatment remains underutilized, as only 2 of 3 patients eligible received treatment.

## Teaser

This cohort study evaluates hepatitis C virus (HCV) treatment rates and patient characteristics associated with treatment in the era of direct-acting antivirals and during the COVID-19 pandemic.

## Introduction

After more than 30 years since the discovery of the hepatitis C virus (HCV), HCV infection remains a global public health concern and continues to be one of the leading causes of liver transplantations in the United States.^1,2^ In 2013 to 2016, approximately 2.4 million people in the US had viremic HCV infection.^3^ About 70% of persons infected with HCV will develop chronic HCV infection, of which 15% to 30% will develop cirrhosis within 20 years if untreated.^1^ Most importantly, HCV is the main contributor to the increase in hepatocellular carcinoma (HCC) incidence in the US over the last several decades.^4,5,6,7,8^

For many years, the standard-of-care treatment for HCV involved injectable interferon. However, interferon-based therapy was lengthy, with considerable adverse effects and poor response rate. As a result, prior to 2014, less than 1 in 6 persons (16%) with HCV infection received antiviral treatment.^9,10,11^

Since 2014, all-oral, short-course, and well-tolerated interferon-free direct-acting antivirals (DAAs) have revolutionized the treatment of HCV, with cure rates approaching 100%.^12,13,14,15,16,17,18^ Unfortunately, substantial barriers hampering the progress of HCV treatment remain, from suboptimal screening, poor disease awareness, and lack of access to care due to limited psychosocial and financial resources, even in high-income countries.^9,19,20,21,22,23,24,25,26,27^ In the US, the opioid epidemic has also increased disease burden among more vulnerable populations and added further barriers to HCV eradication.^28,29^ Finally, the current COVID-19 pandemic likely added further barriers to reaching the World Health Organization (WHO) 2030 HCV elimination goals and widened existing disparities.^30^ However, data on patient characteristics, treatment rates, and factors associated with treatment in the DAA era and during the COVID-19 pandemic are sparse.^31,32^ Therefore, we aimed to characterize patients with HCV in the United States and to determine the treatment rates among those with confirmed viremic infection using a national database covering all regions of the United States from the beginning of the DAA era in 2014 to 2021, which encompasses the COVID-19 pandemic.

## Methods

### Study Design and Study Population

This is a retrospective cohort study of adult patients (≥18 years) with HCV infection using data derived from deidentified Optum’s Clinformatics Data Mart (CDM) Database from 2014 to 2021 through the Population Health Science Center at Stanford University.^33^ CDM is derived from a large adjudicated claims data warehouse for those with private insurance to include those receiving Medicaid who have purchased a supplemental policy. As such, CDM provides longitudinal medical data and prescription drug coverage for 61 million patients as well as results of outpatient laboratory tests from contracted national reference laboratory vendors for approximately 31 million patients. This study was approved by the institutional review board at Stanford University and was given a waiver of consent due to the anonymity of data for data analysis. We followed the Strengthening the Reporting of Observational Studies in Epidemiology (STROBE) reporting guideline.

**Figure 1.**
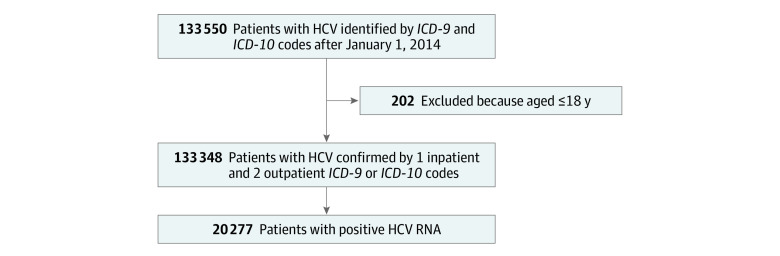
Flowchart of Study Patient Selection *ICD-9* indicates *International Classification of Diseases, Ninth Revision*; *ICD-10*, *International Statistical Classification of Diseases and Related Health Problems, Tenth Revision*; HCV, hepatitis C virus.

First, we searched CDM from January 1, 2014, to March 31, 2021, for patients with HCV infection using the *International Classification of Diseases, Ninth Revision *(*ICD-9*) or *International Statistical Classification of Diseases and Related Health Problems, Tenth Revision *(*ICD-10*) codes for HCV infection (eAppendix in Supplement 1). Eligible patients were adults (aged ≥18 years) who had at least 1 inpatient or 2 outpatient *ICD-9 *(until December 31, 2013) or *ICD-10* (after January 1, 2014) codes for HCV (Figure 1). Viremic HCV infection was defined as those with a positive HCV RNA test. We followed the identified patients and the care they received over the duration of this study.

### Study Variables and Outcomes

Demographic characteristics (age, sex, race and ethnicity, care region, education, occupation, and income), clinical characteristics (clinician type, out-of-pocket expenses, chronic liver disease severity, and comorbidities), and laboratory parameters were obtained within 6 months of the index diagnosis of HCV infection. Race and ethnicity were self-reported and provided by CDM in the following groups: Asian, Black, Hispanic, White, and unknown. Race and ethnicity were part of the demographic characteristics that we observed to help characterize our study cohort as well as to investigate whether there were differences in treatment rates. Geographic location was categorized by US region as defined by the US Census Bureau in the 2010 Census Regions and Divisions of the United States.^34^ Cirrhosis and HCC diagnoses were identified by *ICD-9* and *ICD-10* codes (eAppendix in Supplement 1). Baseline laboratory parameters within 6 months of index HCV diagnosis included alanine aminotransferase, total bilirubin, and creatinine levels. Clinician types were defined by codes provided in the CDM database. We defined *specialist patients* as patients with at least 1 gastroenterologist (GI) or infectious disease (ID) code. Patients without these GI or ID codes were considered primary care physician (PCP)–only patients. Patients receiving care from advance practice practitioners (APPs) were captured using the same methodology via visit codes for nurse practitioners, physician assistants, and clinical nurse specialists.

DAA treatment initiation was defined as having at least 1 DAA prescription, identified via the National Drug Codes for DAA medications (eAppendix in Supplement 1) among those in CDM with pharmaceutical claims. Sustained virologic response (SVR) was defined as having a negative HCV RNA result at any time at least 12 weeks since last DAA prescription. January 1, 2014, to February 29, 2020, was considered the prepandemic time, and the COVID-19 pandemic was considered starting on March 1, 2020, and lasting to the end of the study period on March 31, 2021.

### Statistical Analysis

Categorical variables were reported as counts and percentages, while continuous variables were reported either as mean and SD or median and IQR. Categorical variables were evaluated among groups using Pearson χ^2^ test, while Student *t* test of variance or Wilcoxon rank-sum test was used for continuous variables. We calculated the percentage of patients receiving at least 1 DAA prescription by the total of patients with viremic HCV for the whole cohort and several relevant subgroups by age, sex, race and ethnicity, US region, and time. In addition, we used log-binomial regression with adjustment for relevant clinical factors to estimate prevalence ratios of treatment rates over the study period.

To identify factors associated with HCV treatment initiation, we performed univariable and multivariable logistic regressions to estimate odds ratios (ORs) associating relevant demographic, social, and clinical factors with DAA treatment initiation. Variables included in the multivariable models either had a univariable *P* < .10 or had a potential association with the outcomes of interest by prior reports. All ORs were reported with 95% CIs. Statistical significance was defined as having a 2-tailed *P* < .05. All statistical analyses were conducted using R version 3.5.1 (R Project for Statistical Computing).

## Results

### Characteristics of HCV Patients

In total, we identified 133 348 adult patients with HCV infection (Figure 1). The mean (SD) age of the overall study cohort was 59.7 (12.3) years (Table 1).^35^ Approximately half of the patients were men (79 567 [59.7%]), had a Bachelor’s degree (61 298 [50.2%]), and lived in the South (67 081 [50.4%]). Overall, our sample included 4448 (3.3%) Asian, 24 662 (18.5%) Black, and 74 750 (56.1%) White individuals. Approximately one-third (7818 of 21 810 [35.8%]) were a homemaker or retired, and one-fourth (5654 [25.9%]) were business owners or had held managerial and/or professional positions. However, annual household income in close to half of patients (19 199 of 41 999 [45.7%]) was less than $40 000. Notably, only one-half (71 174 [53.4%]) had at least 1 visit with a GI or ID specialist during the study observation. Approximately 20% of the cohort had cirrhosis and/or HCC (27 346 [20.5%]), and more than one-third had weighted Charlson Comorbidity Index of 5 or greater (46 282 [34.7%]).

**Table 1.** Demographic and Clinical Characteristics of Patients With Hepatitis C, Overall and by Sex

Characteristic	Patients, No. (%)			*P* value
	All (N = 133 348)	Women (n = 53 781)	Men (n = 79 567)	
Age, mean (SD)	59.7 (12.3)	59.8 (13.0)	59.6 (11.7)	<.001
Race and ethnicity				
Asian	4448 (3.3)	1975 (3.7)	2473 (3.1)	<.001
Black	24 662 (18.5)	10 422 (19.4)	14 240 (17.9)	
Hispanic	16 331 (12.2)	6363 (11.8)	9968 (12.5)	
White	74 750 (56.1)	29 984 (55.8)	44 766 (56.3)	
Unknown	13 157 (9.9)	5037 (9.4)	8120 (10.2)	
Care region				
West	29 994 (22.5)	11 850 (22.1)	18 144 (22.9)	<.001
Midwest	20 132 (15.1)	8025 (14.9)	12 107 (15.3)	
Northeast	15 870 (11.9)	6071 (11.3)	9799 (12.3)	
South	67 081 (50.4)	27 741 (51.7)	39 340 (49.6)	
Clinician type				
PCP with APP	29 263 (21.9)	12 372 (23.0)	16 891 (21.2)	<.001
PCP without APP	32 911 (24.7)	12 415 (23.1)	20 496 (25.8)	
GI or ID with APP	43 474 (32.6)	18 305 (34)	25 169 (31.6)	
GI or ID without APP	27 700 (20.8)	10 689 (19.9)	17 011 (21.4)	
Total out-of-pocket expense, median (IQR), $	2214.40 (904.47-4954.11)	2307.81 (985.51-5022.25)	2146.91 (855.03-4910.06)	<.001
Education level				
<12th Grade	886 (0.7)	344 (0.7)	542 (0.7)	.06
High school graduate	47 416 (38.8)	19 044 (38.4)	28 372 (39.1)	
Bachelor’s degree	61 298 (50.2)	24 993 (50.4)	36 305 (50.0)	
>Bachelor’s degree	12 538 (10.3)	5161 (10.4)	7377 (10.2)	
Annual household income, No./total No. (%), $				
<40 000	19 199/41 999 (45.7)	8463/16 691 (50.7)	10 736/25 308 (42.4)	<.001
40 000 to <60 000	7721/41 999 (18.4)	2791/16 691 (16.7)	4930/25 308 (19.5)	
60 000-100 000	8821/41 999 (21.0)	3127/16 691 (18.7)	5694/25 308 (22.5)	
>100 000	6258/41 999 (14.9)	2310/16 691 (13.8)	3948/25 308 (15.6)	
Occupation, No./total No. (%)				
Manager, owner, or professional	5654/21 810 (25.9)	2133/10 676 (19.2)	3521/11 134 (31.6)	<.001
White collar, health care, civil service, military	4450/21 810 (20.4)	2627/10 676 (23.6)	1823/11 134 (17.1)	
Blue collar	3888/21 810 (17.8)	1576/10 676 (14.2)	2312/11 134 (21.7)	
Homemaker or retired	7818/21 810 (35.8)	4798/10 676 (43.1)	3020/11 134 (28.3)	
Mental and psychiatric illnesses^a^	48 250 (36.2)	24 039 (44.7)	24 211 (30.4)	<.001
HIV/AIDS	4591 (3.4)	1089 (2.0)	3502 (4.4)	<.001
Alcohol use disorder	18 667 (14.0)	5108 (9.5)	13 559 (17.0)	<.001
Injection drug use	21 225 (15.9)	8154 (15.2)	13 071 (16.4)	<.001
Smoking	45 951 (34.5)	17 418 (32.4)	28 533 (35.9)	<.001
Homelessness	1451 (1.1)	417 (0.8)	1034 (1.3)	<.001
Weighted Charlson Comorbidity Index^b^				
Mean (SD)	4.0 (3.4)	3.8 (3.3)	4.1 (3.5)	<.001
0	9374 (7.0)	3679 (6.8)	5695 (7.2)	<.001
1-2	50 021 (37.5)	20 836 (38.7)	29 185 (36.7)	
3-4	27 671 (20.8)	11 579 (21.5)	16 092 (20.2)	
≥5	46 282 (34.7)	17 687 (32.9)	28 595 (35.9)	
Liver disease severity				
No cirrhosis or HCC	104 105 (78.1)	43 580 (81.0)	60 525 (76.1)	<.001
Compensated cirrhosis, no HCC	20 983 (15.7)	7741 (14.4)	13 242 (16.6)	
Decompensated cirrhosis and HCC	6363 (4.8)	1909 (3.5)	4454 (5.6)	

Abbreviations: APP, advanced practice practitioner; GI, gastroenterologist; HCC, hepatocellular carcinoma; ID, infectious disease; PCP, primary care physician.

^a^ Mental and psychiatric illnesses include posttraumatic stress disorders, anxiety, panic disorders, depression, bipolar disorder, mania, mood disorders, delusion, psychosis, schizoaffective disorder, schizophrenia, and dementia.

^b^ Weighted Charlson Comorbidity Index adjusts for the number and severity of comorbid diseases according to relative risks presented in Charlson et al.^35^

Compared with women, men were more likely to have an annual household income greater than $100 000 (3948 of 25 308 [15.6%] vs 2310 of 16 691 [13.8%]; *P* < .001) and less likely to be retired or a homemaker (3020 of 10 676 [28.3%] vs 4798 of 11 134 [43.1%]; *P* < .001). Men were also more likely than women to have HIV/AIDS (3502 of 79 567 [4.4%] vs 1089 of 53 781 [2.0%]; *P* < .001) and alcohol use disorder (13 559 [17.0%] vs 5108 [9.5%]; *P* < .001). Additionally, nearly half of women had a mental and psychiatric illness compared with less than one-third of men (24 039 [44.7%] vs 24 211 [30.4%]; *P* < .001).

White, Black, and Hispanic patients were more prevalent in the South and more likely to receive care from GI or ID specialist with APPs, while nearly 40% of Asian patients were from the Western United States (eTable 1 in Supplement 1). The percentage of patients with a bachelor’s degree and graduate education was lowest among Black and Hispanic patients, who also had the lowest annual household income (<$40 000: 5410 of 8143 [66.4%] and 2892 of 5743 [50.4%], respectively). We also found significant differences in liver disease severity across different racial and ethnic groups, with 20% of Hispanic patients (3198 of 15 983) having compensated cirrhosis compared with only 11% to 15% in other groups. Among patients with viremic HCV, approximately one-third (6956 of 20 277 [34.3%]) did not have an encounter with a GI or ID specialist (for any reason) during the entire study observation period (eTable 2 in Supplement 1).

### DAA Treatment Rate and SVR Rate Among Patients With Viremic Hepatitis C

**Figure 2.**
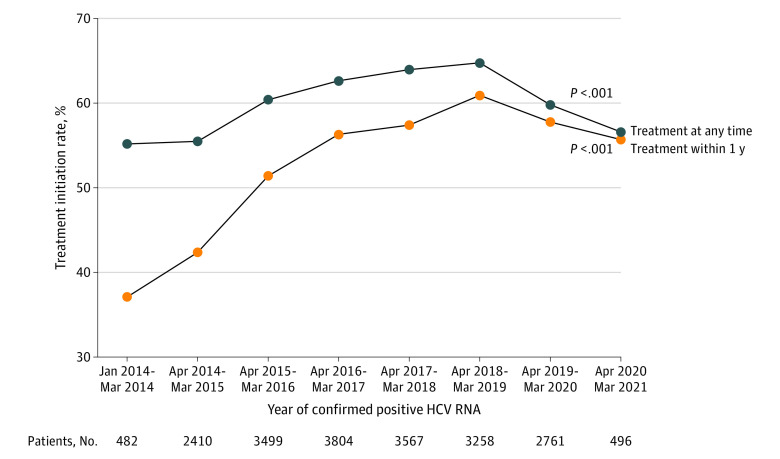
Trend of the Treatment Rate of Patients With Viremic Hepatitis C Virus (HCV) Over Time

Of the total cohort, 38 180 patients (28.6%) had an HCV RNA test result at baseline, and 20 277 of these (53.1%) had a positive HCV RNA result. Of the 20 277 patients with viremic HCV, 13 214 (65.2%; 95% CI, 64.5%-65.8%) received DAA treatment, with highest rate (5342 of 7278 [73.4%; 95% CI, 72.4%-74.4%]) among those who received care from GI or ID with APP, followed by GI or ID without APP (4104 of 6043 [67.9%; 95% CI, 66.7%-69.1%]), PCP with APP (1908 of 3269 [58.4%; 95% CI, 53.7%-60.1%]), and the lowest rate (1860 of 3687 [50.4%; 95% CI, 48.8%-52.1%]) among those seen by a PCP without APP (*P* < .001) (Table 2). Among 3593 patients with compensated cirrhosis, 2747 (76.5%; 95% CI, 75.1%-77.8%) were treated, compared with 482 of 860 (56.0%; 95% CI, 52.7%-59.4%) of those with hepatic decompensation and/or HCC, while the treatment rate among patients with viremic HCV without cirrhosis was 63.0% (95% CI, 62.3%-63.8% [9913 of 15 723]). Additionally, just more than half of patients with a history of injection drug use received DAA treatment (1822 of 3336 [54.6%; 95% CI, 52.9%-56.3%]). Notably, the DAA treatment rate within 1 year of a positive HCV RNA test initially increased by almost one-third (27%) from 38.0% in January 2014 to 64.8% in March 2019 (Figure 2). In fact, after adjusting for sex, race and ethnicity, and age, the treatment rate in 2018 was 0.5 times greater (adjusted prevalence ratio, 1.50; 95% CI, 1.42-1.59) than the rate observed in 2014 (eTable 3 in Supplement 1). However, the treatment rate declined in recent years, with only 61.2% receiving treatment between April 2019 and March 2020 compared with 64.8% between April 2018 and March 2019 (*P* < .001) and further declined to less than 60% between April 2020 to March 2021 (*P* < .001). In addition, during the COVID-19 pandemic, only 496 patients with viremic HCV were identified between April 2020 to March 2021, a sizable decrease compared with the number of viremic patients identified in the prepandemic period (2761 between April 2019 and March 2020; 3258 between April 2018 and March 2019) (Figure 2).

**Table 2.** Treatment Rate of Patients With Positive HCV RNA

Group	Total No.	Treatment initiation rate, No. (%) [95% CI]	*P* value
All patients with positive HCV RNA results	20 277	13 214 (65.2) [64.5-65.8]	NA
Sex			
Male	12 594	8134 (64.6) [63.8-65.4]	.03
Female	7683	5080 (66.1) [65.1-67.2]	
Race and ethnicity			
Asian	461	293 (63.6) [59.2-68]	<.001
Black	3913	2583 (66.0) [64.5-67.5]	
Hispanic	2606	1758 (67.5) [65.7-69.3]	
White	11 454	7475 (65.3) [64.4-66.1]	
Unknown	1843	1105 (60.0) [57.7-62.2]	
Liver disease severity			
No cirrhosis or HCC	15 723	9913 (63.0) [62.3-63.8]	<.001
Compensated cirrhosis, no HCC	3593	2747 (76.5) [75.1-77.8]	
Decompensated cirrhosis and HCC	860	482 (56.0) [52.7-59.4]	
Care regions			
West	5217	3360 (64.4) [63.1-65.7]	<.001
Midwest	1713	1041 (60.8) [58.5-63.1]	
Northeast	1786	1144 (64.1) [61.8-66.3]	
South	11 530	7657 (66.4) [65.5-67.3]	
Clinician type			
PCP with APP	3269	1908 (58.4) [56.7-60.1]	<.001
PCP without APP	3687	1860 (50.4) [48.8-52.1]	
GI or ID with APP	7278	5342 (73.4) [72.4-74.4]	
GI or ID without APP	6043	4104 (67.9) [66.7-69.1]	
Education			
<12th Grade	162	100 (61.7) [54.2-69.2]	.03
High school graduate	7431	4795 (64.5) [63.4-65.6]	
Bachelor’s degree	9365	6239 (66.6) [65.7-67.6]	
>Bachelor’s degree	1743	1150 (66.0) [63.8-68.2]	
Annual household income, $			
<40 000	2993	1741 (58.2) [56.4-59.9]	.78
40 000 to <60 000	1281	731 (57.1) [54.4-59.8]	
60 000-100 000	1428	843 (59.0) [56.5-61.6]	
>100 000	992	579 (58.4) [55.3-61.4]	
Mental and psychiatric illnesses^a^			
Yes	6705	4172 (62.2) [61.1-63.4]	.004
No	13 572	9042 (66.6) [65.8-67.4]	
HIV/AIDS			
Yes	631	398 (63.1) [59.3-66.8]	.60
No	19 646	12 816 (65.2) [64.6-65.9]	
Alcohol use disorder			
Yes	2852	1584 (55.5) [53.7-57.4]	<.001
No	17 425	11 630 (66.7) [66-67.4]	
Injection drug use			
Yes	3336	1822 (54.6) [52.9-56.3]	<.001
No	16 941	11 392 (67.2) [66.5-68]	

Abbreviations: APP, advanced practice practitioner; GI, gastroenterologist; HCC, hepatocellular carcinoma; HCV, hepatitis C virus; ID, infectious disease; NA, not applicable; PCP, primary care physician.

^a^ Mental and psychiatric illnesses include posttraumatic stress disorders, anxiety, panic disorders, depression, bipolar disorder, mania, mood disorders, delusion, psychosis, schizoaffective disorder, schizophrenia, and dementia.

Among the 6634 patients who received DAA treatment and had SVR data, the overall cure rate was excellent, with 6456 (97.3%; 95% CI, 96.9%-97.7%) achieving SVR, while the SVR rates were slightly lower, at 221 of 244 (90.6%; 95% CI, 86.9%-94.2%) and 769 of 809 (95.1%; 95% CI, 93.6%-96.5%) among those with HIV/AIDS coinfection and those with a history of injection drug use, respectively (eTable 4 in Supplement 1). There was a significant though small difference in the SVR rates among different racial and ethnic groups, ranging from 1254 of 1301 (96.4%; 95% CI, 95.4%-97.4%) among Black patients to 951 of 969 (98.1%; 95% CI, 97.3%-99.0%) in the Hispanic group (*P* = .04), while there were no significant differences in SVR rates among patients receiving care in different regions, with or without GI or ID and/or APP, as well as by education and annual household income levels.

### Factors Associated With Initiating DAA Treatment

**Figure 3.**
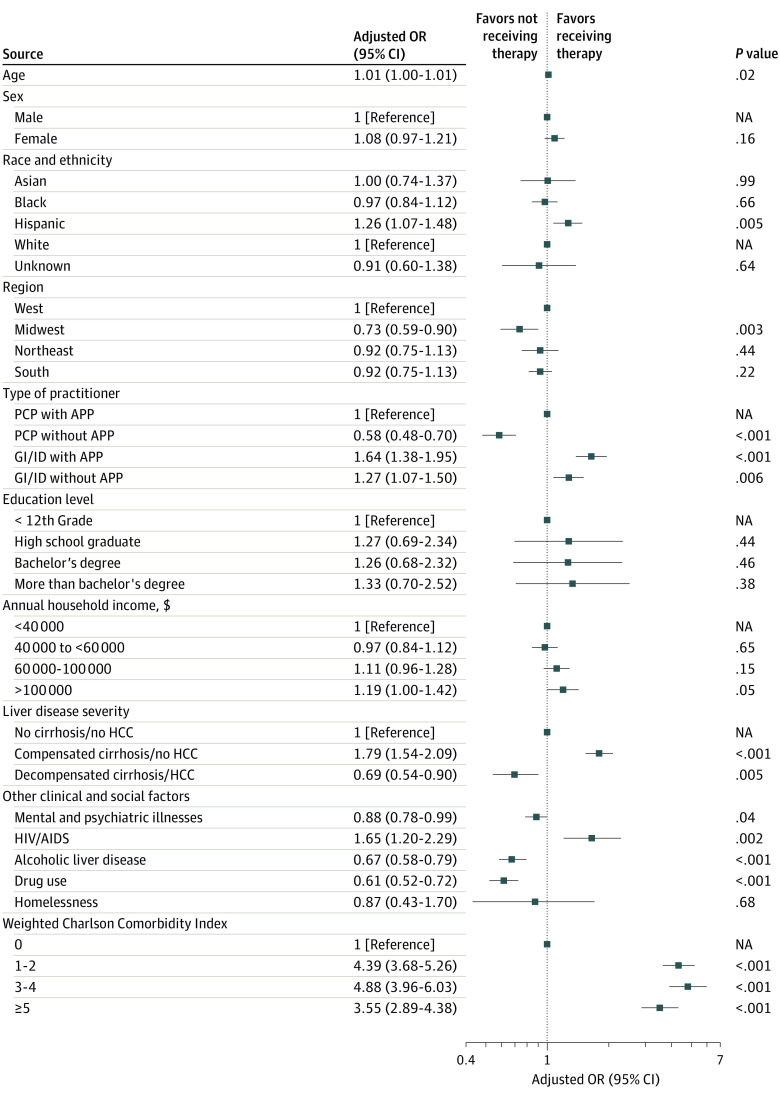
Factors Associated With Hepatitis C Virus Antiviral Treatment Initiation APP indicates advanced practice practitioner; GI, gastroenterologist; ID, infectious disease specialist; NA, not applicable; OR, odds ratio; PCP, primary care physician.

On multivariable logistic regression analysis (Figure 3), compared with patients who received care from a PCP with APP, those who received care from a GI or ID specialist with an APP were 65% more likely to receive treatment (adjusted OR [aOR], 1.64; 95% CI, 1.38-1.95; *P* < .001) while patients treated by GI or ID specialists without an APP were only approximately 30% more likely to receive treatment (aOR, 1.27; 95% CI, 1.07-1.50; *P* = .006). Meanwhile, patients receiving only PCP care without an APP were 42% less likely to receive treatment compared with patients seeing a PCP with an APP (aOR, 0.58; 95% CI, 0.48-0.70; *P* < .001). Additionally, patients receiving care in the Midwest of were approximately 30% less likely to receive treatment compared with those in the West (aOR, 0.73; 95% CI, 0.59-0.90; *P* = .003). Patients with decompensated cirrhosis and/or HCC were 30% less likely to receive treatment compared with patients without cirrhosis (aOR, 0.69; 95% CI, 0.54-0.90; *P* = .005).

## Discussion

In this nationwide US study of insured HCV patients conducted between 2014 and 2021, we found suboptimal treatment rates for patients with viremic HCV infection, especially since the beginning of the COVID-19 pandemic. We found a high cure rate of approximately 97% among patients treated with DAAs, regardless of what kind of clinician provided care. However, less than two-thirds of all patients with viremic HCV received DAA treatment. Even among patients with compensated cirrhosis, only 77% received treatment. Alarmingly, only about half of patients who had decompensation and/or HCC received treatment. Patients who had at least 1 visit with a GI or ID specialist and APP had significantly greater odds of receiving treatment compared with those who received care from a PCP with an APP. In addition, compared with patients who received care in the Western region of the United States, those from the Midwest were much less likely to receive treatment. These findings highlight the ongoing suboptimal treatment rate even among patients who were insured in the United States, requiring aggressive measures to increase screening for HCV.

Because of the demonstrated advantages and benefits of the new DAA treatment regimens, it is expected that there would be an initial surge in treatment initiation rates as patients who were more motivated, had more severe illness, and had better insurance with access to a hepatologist, GI, or ID specialist would likely seek treatment and that the treatment initiation would dwindle over time as the initial HCV cohort were cured.^36^ In fact, this is what we found: the rate of treatment increased by one-third from 2014 to 2018 and then began to decrease after 2018. Additionally, the DAA treatment rate as well as the number of viremic HCV diagnoses further plummeted since the onset of the COVID-19 pandemic in early 2020, and this is likely because of shelter-in-place orders, disrupted health care patterns, and shifting of resources to address the more immediate COVID-19–related crisis. If the initial treatment trajectory had continued and the number of new HCV infections continued, then the United States may have been on the way to achieve viral elimination by 2030, as called for by the WHO.^37^ However, given the declining treatment rates, the declining number of viremic HCV diagnoses beginning in 2019 (before the pandemic), and the rise in new HCV infection related to the opioid crisis,^38,39^ this encouraging trend has most likely been reversed. Therefore, we suggest an urgent call to action is needed to reverse these trends and get the United States back on the road to HCV elimination by 2030. The fight against chronic HCV infection cannot be tabled, as a 1-year delay in HCV diagnosis and treatment can cause 72 000 excess deaths from HCV worldwide.^40^

We also found significant differences in the distribution of HCV infection across US regions, with half of the study cohort receiving care in the South. This finding may also reflect the impact of the opioid epidemic in igniting a dramatic increase of HCV cases, as most new HCV cases have been observed in persons who inject drugs and there has been a steep 364% increase in acute HCV cases among young adults in 4 Appalachian states in the South between 2006 and 2012.^38,39^ We also found that patients who received care in the Midwest were 30% less likely to be associated with receiving DAA treatment compared with those receiving care in the West, after adjusting for race and ethnicity, income, education level, and liver disease severity.

Another important observation in the current study is the 30% lower likelihood of receiving DAA therapy among patients with decompensated cirrhosis and/or HCC despite the finding that among patients with decompensated cirrhosis or HCC who received treatment, 95% achieved SVR. These results highlight the need to expand awareness that patients with advanced disease should be prioritized to receive treatment and not the reverse, as in addition to our study, other multiple large and well-controlled studies have shown improved liver function and survival with HCV cure with DAA in patients with decompensated liver cirrhosis and/or HCC.^41,42,43,44^ One potential reason for the lower treatment rates among these patients could be lack of financial and social support to seek care given that these patients may be too sick to navigate the medical system and/or drive themselves to care appointments. Another reason may be clinician hesitation to treat patients with end-stage liver disease or HCC due to concerns of adverse effects, lower treatment response, and in the case of HCC, fear of higher risk of HCC incidence and recurrence, as reported by early case reports and case series.^45^ However, several other studies have reported excellent safety profiles and high cure rates with DAA even in patients with advanced disease, including HCC.^42,46,47,48,49,50^

Finally, although we did not directly measure barriers to treatment, cost of treatment may be a barrier even among those with insurance. Specifically, the amount of total out-of-pocket expense in relation to the average annual household income for the patients in this study can be significant. For example, almost half of the cohort had an average annual household income of less than $40 000, and the median out-of-pocket expense was $2214, which is approximately 5% of the annual household income if the denominator is at the top of the scale ($39 900) and could be as high as 15% if the denominator is at the low end of the scale ($15 080, based on the minimum federal hourly wage, which has been stagnant at $7.25 since 2009).^51^ A recent survey conducted among adults in the United States found that due to high costs of medications (to include copayments), 13% of respondents said they would skip buying their medications, 9% would cut their pills, 3% would buy from a foreign country, 3% would buy on the black market, and 50% would switch to a generic version of the drug.^52^ However, since generic DAAs are not available in the United States, this is not a viable option and suggests that patients may not have purchased their medications. As such, research into costs and other barriers is needed.

### Strengths and Limitations

The strength of our study is the large sample size with a diverse racial and ethnic patient population from different regions of the United States, which makes our findings more generalizable than local institutional cohorts. Our study also uses HCV RNA to confirm HCV infection and assess the SVR rate, which provides a more accurate assessment of the current patient characteristics and treatment rates compared with *ICD*-*9* and *ICD-10* codes alone. We identified factors associated with higher odds of receiving DAA treatment, such as geographical location and access to specialists and APPs, that help inform efforts to fill the current gaps in HCV treatment cascade in the United States. Lastly, we identified another important disparity in treatment rates for patients who most needed treatment, ie, those with decompensated cirrhosis and/or HCC, so further patient and clinician education are needed.

This study also has limitations. Our study cohort included only insured persons, which might lead to a potential selection bias for patients with better access to care. However, given the low treatment rate even among those who are better insured, our findings are important in demonstrating that the treatment rate might be even worse for those who are uninsured or underinsured. Our definition for DAA treatment initiation was liberal and could overestimate the true treatment rate because receipt of a prescription does not necessarily mean the patient took the medication or adhered to the full course of treatment. However, even with such simple and loose definition, the treatment rate was still suboptimal, further highlighting the important care gaps and the magnitude of the problem shown in this study. Large claim databases can be subject to miscoding. To circumvent this issue, we included only patients with positive HCV RNA to calculate the treatment rate. Furthermore, patients with decompensated cirrhosis could have been treated by a specialist outside their insurance network; however, the treatment claim should still have been available to us for analysis. Additionally, we acknowledge that there may be patients lost to follow-up; however, if a patient changes to another private insurance plan, they most likely stayed within the database, allowing us to continue to follow their care and outcomes. We also investigated treatment rates during the first year after diagnosis as well as any time following diagnosis as a confirmation method and found that the rates for any time after diagnosis were slightly higher than in the first year. This result does help to show that our patient population may be stable without a lot of turnovers.

## Conclusions

Despite having private health insurance, the treatment rate for HCV infection with DAA remains suboptimal across the United States. Additionally, treatment rates since 2019 have declined. Future efforts must be made to increase access to DAA treatment in community clinics and drug-dependency clinics for persons who inject drugs as well as improving the referral pipeline to specialists especially for individuals with HCV cirrhosis and HCC.

## Notes

Supplement 1. **eAppendix. ***ICD *Codes **eTable 1. **Demographic and Clinical Characteristics of Patients With Hepatitis C by Race and Ethnicity **eTable 2. **Demographic and Clinical Characteristics of Patients With Viremic Hepatitis C **eTable 3. **Prevalence Ratios for DAA Treatment Rate Over Study Period **eTable 4. **Proportion of Patients With Viremic HCV Receiving Treatment and Achieving SVR

Supplement 2.
Data Sharing Statement
