# Supplementary material for: Characteristics and Treatment Rate of Patients With Hepatitis C Virus Infection in the Direct-Acting Antiviral Era and During the COVID-19 Pandemic in the United States
Source: JAMA Netw Open. 2022 Dec 7;5(12):e2245424. doi: 10.1001/jamanetworkopen.2022.45424 (PMC9856330; doi:10.1001/jamanetworkopen.2022.45424)
Supplement: Supplement 1. — eAppendix. ICD Codes eTable 1. Demographic and Clinical Characteristics of Patients With Hepatitis C by Race and Ethnicity eTable 2. Demographic and Clinical Characteristics of Patients With Viremic Hepatitis C eTable 3. Prevalence Ratios for DAA Treatment Rate Over Study Period eTable 4. Proportion of Patients With Viremic HCV Receiving Treatment and Achieving SVR [file jamanetwopen-e2245424-s001.pdf]

# Supplemental Online Content

Nguyen VH, Kam L, Yeo YH, et al. Characteristics and treatment rate of patients with hepatitis C virus infection in the direct-acting antiviral era and during the COVID-19 pandemic in the United States. *JAMA Netw Open.* 2022;5(12):e2245424. doi:10.1001/jamanetworkopen.2022.45424

**eAppendix.** *ICD* Codes

**eTable 1.** Demographic and Clinical Characteristics of Patients With Hepatitis C by Race and Ethnicity

**eTable 2.** Demographic and Clinical Characteristics of Patients With Viremic Hepatitis C

**eTable 3.** Prevalence Ratios for DAA Treatment Rate Over Study Period

**eTable 4.** Proportion of Patients With Viremic HCV Receiving Treatment and Achieving SVR

This supplemental material has been provided by the authors to give readers additional information about their work.

© 2022 Nguyen VH et al. *JAMA Network Open.*

**eAppendix.** ***ICD*** **Codes**

***ICD codes for HCV infection:*** 070.22, 070.32, B18.1, 070.70, 070.71, v0262, B19.20, B19.21
***ICD codes for liver cirrhosis and HCC:*** 571.2, 571.5, 571.6, K70.30, K74.0, K74.60, K74.69, K74.3, K74.4, K74.5, 572.3, 789.2, 456.1, 456.21, K76.6, R16.1, I85.00, I85.10, 155.0, C22.0, C22.8
***National Drug Codes for DAA medications:***
**Simprevir**: 59676022528, 59676022507
**Paritaprevir**: 00074006301, 00074006328, 00074308228, 00074309301, 00074309328
**Asunaprevir**: 11014017901
**Ledipasvir**: 61958180101, 72626260101
**Ombitasvir**: 00074006301, 00074006328, 00074308228, 00074309301, 00074309328
Sofosbuvir: 61958150101, 61958180101, 61958220101, 61958240101, 72626260101, 72626270101
**Sovaldi**: 61958150101
**Dasabuvir**: 00074006301, 00074006328, 00074309301, 00074309328
**Glecaprevir**: 00074262501, 00074262528, 00074262556
**Pibrentasvir**: 00074262501, 00074262528, 00074262556
**Velpatsvir**: 61958220101, 61958240101, 72626270101
**Voxilaprevir**: 61958240101
**Grazoprevir**: 00006307402
**Elbasvir**: 00006307402
**Ritonavir**: 00054040713, 00074006301, 00074006328, 00074052260, 00074194063, 00074308228, 00074309301, 00074309328, 00007433330, 00074339930, 00074395646, 00074679922, 00074679930, 00527194748, 31722059712, 31922059730, 50090116200, 53808027601, 53808111901, 55289094712, 60687036425, 60687042025, 65162006106, 65162006132, 65862068701, 65862068705, 65862068730, 65862068799, 68382069606, 70518009100, 70518009101, 70518009102, 70518108600, 70518151900, 70518200700

© 2022 Nguyen VH et al. *JAMA Network Open.*

**eTable 1.** Demographic and Clinical Characteristics of Patients With Hepatitis C by Race and Ethnicity

| | White (N= 74,750) | Black (N= 24,662) | Asian (N= 4,448) | Hispanic (N= 16,331) | Unknown[b] (N=13,157) | *P*-value |
|---|---|---|---|---|---|---|
| **Mean age (SD)** | 58.7 (12.6) | 62 (10.2) | 60.4 (14.2) | 59.8 (12.7) | 60.2 (11.9) | <0.001 |
| **Male (%)** | 44,766 (59.9) | 14,240 (57.7) | 2,473 (55.6) | 9,968 (61) | 8,120 (61.7) | <0.001 |
| **Care region (%)** | | | | | | <0.001 |
| West | 17,785 (23.8) | 1,948 (7.9) | 1822 (41.0) | 4,944 (30.4) | 3,495 (26.6) | |
| Midwest | 13,033 (17.5) | 4,006 (16.3) | 381 (8.6) | 979 (6.0) | 1,733 (13.2) | |
| Northeast | 9,163 (12.3) | 2,445 (9.9) | 799 (18.0) | 2,162 (13.3) | 1,301 (9.9) | |
| South | 34,626 (46.4) | 16,232 (65.9) | 1,440 (32.4) | 8,190 (50.3) | 6,593 (50.2) | |
| **Provider type (%)** | | | | | | <0.001 |
| PCP with APP | 17,048 (22.8) | 5,209 (21.1) | 648 (14.6) | 3,223 (19.7) | 3,135 (23.8) | |
| PCP without APP | 17,223 (23.0) | 5,433 (22.0) | 1,665 (37.4) | 4,549 (27.9) | 4,041 (30.7) | |
| GI/ID with APP | 25,204 (33.7) | 8,834 (35.8) | 946 (21.3) | 5,008 (30.7) | 3,482 (26.5) | |
| GI/ID without APP | 15,275 (20.4) | 5,186 (21.0) | 1,189 (26.7) | 3,551 (21.7) | 2,499 (19.0) | |
| **Total out-of-pocket expense (Median, IQR)** | 2,538.32, 1,052.65-5,640.26 | 2,203.41, 969.42-4,390.83 | 1,749.51, 603.43-4,202.49 | 1,891.72, 765.71-4,243.56 | 1,315.63, 500.20-3,201.97 | <0.001 |
| **Education Level (%)** | | | | | | <0.001 |
| < 12th grade | 257 (0.3) | 79 (0.3) | 35 (0.8) | 507 (3.1) | | |
| High school graduate | 24,397 (32.7) | 13,464 (54.7) | 1,120 (25.2) | 7,839 (48.1) | | |
| Bachelor's degree | 41,075 (55.1) | 9,824 (39.9) | 2,296 (51.6) | 6,910 (42.4) | | |
| > Bachelor's degree | 8,879 (11.9) | 1,268 (5.1) | 995 (22.4) | 1,039 (6.4) | | |
| **Annual Household Income (%)** | | | | | | <0.001 |
| < $40,000 | 10,147 (39.7) | 5,410 (66.4) | 480 (27.3) | 2,892 (50.4) | 270 (33.8) | |
| $40,000- <$60,000 | 4,624 (18.1) | 1,418 (17.4) | 316 (18.0) | 1,220 (21.2) | 143 (17.9) | |
| $60,000 - $100,000 | 6,121 (24.0) | 961 (11.8) | 477 (27.2) | 1,067 (18.6) | 195 (24.4) | |
| > $100,000 | 4,665 (18.3) | 354 (4.3) | 483 (27.5) | 564 (9.8) | 192 (24.0) | |
| **Occupation (%)** | | | | | | |
| Manager/Owner/ Professional | 4,062 (26.5) | 804 (22.4) | 181 (34.0) | 509 (25.6) | 98 (27.0) | <0.001 |
| White collar/Health/ Civil Service/ Military | 3,081 (20.1) | 735 (20.5) | 124 (23.3) | 416 (20.9) | 94 (25.9) | |

© 2022 Nguyen VH et al. *JAMA Network Open.*

| | | | | | | |
|---|---|---|---|---|---|---|
| Blue Collar | 2,694 (17.6) | 598 (16.6) | 124 (23.3) | 410 (20.6) | 62 (17.1) | |
| Homemaker/ Retired | 5,494 (35.8) | 1,456 (40.5) | 103 (19.4) | 656 (32.9) | 109 (30.0) | |
| **Mental and psychiatric illnesses[a] (%)** | 29,692 (39.7) | 7,749 (31.4) | 735 (16.5) | 5,408 (33.1) | 4,666 (15.9) | <0.001 |
| **HIV/AIDS (%)** | 1,931 (2.6) | 1,556 (6.3) | 79 (1.8) | 678 (4.2) | 447 (1.5) | <0.001 |
| **Alcohol use disorder (%)** | 10,771 (14.4) | 3,270 (13.3) | 245 (5.5) | 2,398 (14.7) | 1,983 (6.8) | <0.001 |
| **Injection drug use (%)** | 12,955 (17.3) | 3,695 (15.0) | 220 (4.9) | 2,244 (13.7) | 2,111 (7.2) | <0.001 |
| **Smoking (%)** | 27,878 (37.3) | 9,054 (36.7) | 616 (13.8) | 4,071 (24.9) | 4,333 (14.8) | <0.001 |
| **Homelessness (%)** | 788 (1.1) | 301 (1.2) | 15 (0.3) | 134 (0.8) | 213 (0.7) | <0.001 |
| **Weighted Charlson's comorbidity index[c] Mean (SD)** | 3.7 (3.3) | 4.8 (3.8) | 3.3 (3.1) | 4.2 (3.5) | 3.9 (3.2) | <0.001 |
| 0 (%) | 5,881 (7.9) | 1,430 (5.8) | 367 (8.3) | 1,131 (6.9) | 565 (4.3) | <0.001 |
| 1-2 (%) | 29,936 (40.0) | 7,196 (29.2) | 2,057 (46.2) | 5,532 (33.9) | 5,300 (40.3) | |
| 3-4 (%) | 15,452 (20.7) | 5,166 (20.9) | 861 (19.4) | 3,385 (20.7) | 2,807 (21.3) | |
| ≥ 5 (%) | 23,481 (31.4) | 10,870 (44.1) | 1,163 (26.1) | 6,283 (38.5) | 4,485 (34.1) | |
| **Liver disease severity (%)** | | | | | | |
| No cirrhosis/no HCC | 58,884 (79.9) | 19,890 (81.4) | 3,646 (83.2) | 11,852 (74.2) | 9,833 (76.0) | <0.001 |
| Compensated cirrhosis/no HCC | 11,501 (15.6) | 3,484 (14.3) | 495 (11.3) | 3,198 (20.0) | 2,305 (17.8) | |
| Decompensated cirrhosis/HCC | 3,330 (4.5) | 1,060 (4.3) | 241 (5.5) | 933 (5.8) | 799 (6.2) | |

Mean (SD) or N(%).

HCV, hepatitis C virus; SD, standard deviation; GI, gastroenterologist; ID, infectious disease; APP, advanced practice provider; HIV, human immunodeficiency viruses; AIDS, acquired immunodeficiency syndrome; HCC, hepatocellular carcinoma; IQR, interquartile range

[a]Mental and psychiatric illnesses include post-traumatic stress disorders, anxiety, panic disorders, depression, bipolar disorder, mania, mood disorders, delusion, psychosis, schizoaffective disorder, schizophrenia, dementia

[b]Education levels for unknown race and ethnicity were not available for presentation due to Stanford PHS policy of data suppression if one cell contains less than 10 patients.

[c]Weighted Charlson's comorbidity index adjusts for the number and severity of comorbid diseases according to relative risks presented in Charlson et al. A new method of classifying prognostic comorbidity in longitudinal studies: development and validation. J Chronic Dis. 1987;40(5)373-83.

© 2022 Nguyen VH et al. *JAMA Network Open.*

**eTable 2.** Demographic and Clinical Characteristics of Patients With Viremic Hepatitis C

| | **Viremic HCV patients (N= 20,277)** |
|---|---|
| **Mean age (SD)** | 57.9 (12.8) |
| **Male (%)** | 12,594 (62.1) |
| **Race and ethnicity (%)** | |
| White | 11,454 (56.5) |
| Black | 3,913 (19.3) |
| Asian | 461 (2.3) |
| Hispanic | 2,606 (12.9) |
| Unknown | 1,843 (9.1) |
| **Care region (%)** | |
| West | 5,217 (25.8) |
| Midwest | 1,713 (8.5) |
| Northeast | 1,786 (8.8) |
| South | 11,530 (56.9) |
| **Provider type (%)** | |
| PCP with APP | 3,269 (16.1) |
| PCP without APP | 3,687 (18.2) |
| GI/ID with APP | 7,278 (35.9) |
| GI/ID without APP | 6,043 (29.8) |
| **Total out-of-pocket expense (Median, IQR)** | (2,717.30, 1,082.82-6,283.74) |
| **Education Level (%)** | |
| < 12th grade | 162 (0.9) |
| High school graduate | 7,431 (39.7) |
| Bachelor's degree | 9,365 (50.1) |
| > Bachelor's degree | 1,743 (9.3) |
| **Annual Household Income (%)** | |
| < $40,000 | 2,993 (44.7) |
| $40,000- <$60,000 | 1,281 (19.1) |
| $60,000 - $100,000 | 1,428 (21.3) |
| > $100,000 | 992 (14.8) |
| **Occupation (%)** | |
| Manager/Owner/Professional | 802 (26.8) |
| White collar/Health/ Civil Service/ Military | 612 (20.4) |
| Blue Collar | 569 (19.0) |
| Homemaker/ Retired | 1,013 (33.8) |
| **Mental and psychiatric illnesses[a] (%)** | 6,705 (33.1) |
| **HIV/AIDS (%)** | 631 (3.1) |
| **Alcohol use disorder (%)** | 2,852 (14.1) |
| **Injection drug use (%)** | 3,336 (16.5) |
| **Smoking (%)** | 7,405 (36.5) |
| **Homelessness (%)** | 167 (0.8) |

© 2022 Nguyen VH et al. *JAMA Network Open.*

| | |
|---|---|
| **Weighted Charlson's comorbidity index[b] (Mean, SD)** | 3.5 (3.2) |
| 0 (%) | 1,328 (6.5) |
| 1-2 (%) | 8,996 (44.4) |
| 3-4 (%) | 4,144 (20.4) |
| ≥ 5 (%) | 5,809 (28.6) |
| **Liver disease severity (%)** | |
| No cirrhosis/no HCC | 15,723 (77.9) |
| Compensated cirrhosis/no HCC | 3,593 (17.8) |
| Decompensated cirrhosis/HCC | 860 (4.3) |
| **Mean ALT (IU/mL)** | 74.2 (103.4) |
| **Mean total bilirubin (mg/dL)** | 0.66 (0.75) |
| **Mean creatinine (mg/dL)** | 0.95 (0.51) |

Mean (SD) or N(%).

HCV, hepatitis C virus; SD, standard deviation; GI, gastroenterologist; ID, infectious disease; APP, advanced practice provider; HIV, human immunodeficiency viruses; AIDS, acquired immunodeficiency syndrome; HCC, hepatocellular carcinoma; IQR, interquartile range; ALT, alanine aminotransferase

[a]Mental and psychiatric illnesses include post-traumatic stress disorders, anxiety, panic disorders, depression, bipolar disorder, mania, mood disorders, delusion, psychosis, schizoaffective disorder, schizophrenia, dementia

[b]Weighted Charlson's comorbidity index adjusts for the number and severity of comorbid diseases according to relative risks presented in Charlson et al. A new method of classifying prognostic comorbidity in longitudinal studies: development and validation. J Chronic Dis. 1987;40(5)373-83

© 2022 Nguyen VH et al. *JAMA Network Open.*

**eTable 3.** Prevalence Ratios for DAA Treatment Rate Over Study Period[a]

| | 2014 vs. 2015 | | 2014 vs. 2016 | | 2014 vs. 2017 | | 2014 vs. 2018 | | 2018 vs. 2019 | | 2018 vs. 2020 | |
|---|---|---|---|---|---|---|---|---|---|---|---|---|
| | Crude | Adjusted[b] | Crude | Adjusted[b] | Crude | Adjusted[b] | Crude | Adjusted[b] | Crude | Adjusted[b] | Crude | Adjusted[b] |
| Treatment within 1 year | 1.23 (1.17-1.30) | 1.23 (1.16-1.3) | 1.36 (1.29-1.43) | 1.35 (1.28-1.42) | 1.39 (1.31-1.46) | 1.38 (1.31-1.46) | 1.48 (1.4-1.55) | 1.5 (1.42-1.59) | 0.95 (0.91-0.98) | 0.94 (0.9-0.98) | 0.91 (0.84-0.98) | 0.88 (0.81-0.96) |
| Treatment at any time | 1.09 (1.05-1.14) | 1.1 (1.05-1.14) | 1.14 (1.09-1.18) | 1.13 (1.09-1.18) | 1.16 (1.12-1.21) | 1.16 (1.12-1.21) | 1.18 (1.13-1.23) | 1.2 (1.15-1.25) | 0.92 (0.89-0.95) | 0.91 (0.88-0.95) | 0.87 (0.8-0.93) | 0.84 (0.78-0.92) |

DAAs, direct acting antivirals

[a]Each year is defined from April to March of the next year (e.g., 2014: April 2014 – March 2015)

[b]Adjusted for age, race, and sex

© 2022 Nguyen VH et al. *JAMA Network Open.*

**eTable 4.** Proportion of Patients With Viremic HCV Receiving Treatment and Achieving SVR

| | SVR rate (%) | 95% CI | *P*-value |
|---|---|---|---|
| All (+) HCV RNA patients with available SVR (N=6,634) | 6,456 (97.3) | 96.9 - 97.7 | |
| **Sex** | | | |
| Male (N=3,986) | 3,869 (97.1) | 97.1 - 98.3 | 0.12 |
| Female (N=2,648) | 2,587 (97.7) | 96.5 - 97.6 | |
| **Race and ethnicity** | | | |
| Asian (N=175) | 169 (96.6) | 93.9 - 99.3 | 0.04 |
| Black (N=1,301) | 1,254 (96.4) | 95.4 - 97.4 | |
| Hispanic (N=969) | 951 (98.1) | 97.3 - 99 | |
| White (N=3,718) | 3,618 (97.3) | 96.8 - 97.8 | |
| Unknown (N=471) | 464 (98.5) | 97.4 - 99.6 | |
| **Liver disease severity** | | | |
| No cirrhosis/no HCC (N=4,892) | 4,767 (97.4) | 97 - 97.9 | 0.52 |
| Compensated cirrhosis/no HCC (N=1,476) | 1,433 (97.1) | 96.2 - 97.9 | |
| Decompensated cirrhosis/HCC (N=222) | 213 (95.9) | 93.4 - 98.5 | |
| **Care regions** | | | |
| West (N=1,828) | 1,786 (97.7) | 97 - 98.4 | 0.64 |
| Midwest (N=415) | 404 (97.3) | 95.8 - 98.9 | |
| Northeast (N=580) | 562 (96.9) | 95.5 - 98.3 | |
| South (N=3,804) | 3,697 (97.2) | 96.7 - 97.7 | |
| **Provider type** | | | |
| PCP with APP (N=889) | 873 (98.2) | 97.3 - 99.1 | 0.25 |
| PCP without APP (N=897) | 871 (97.1) | 96 - 98.2 | |
| GI/ID with APP (N=2,943) | 2,855 (97.0) | 96.4 - 97.6 | |
| GI/ID without APP (N=1,905) | 1,857 (97.5) | 96.8 - 98.2 | |
| **Education Level** | | | |
| < 12th grade (N=61) | 60 (98.4) | 95.2 - 100 | 0.91 |
| High school graduate (N=2,355) | 2,288 (97.2) | 96.5 - 97.8 | |
| Bachelor's degree (N=3,207) | 3,117 (97.2) | 96.6 - 97.8 | |
| > Bachelor's degree (N=641) | 625 (97.5) | 96.3 - 98.7 | |
| **Annual Household Income** | | | |
| < $40,000 (N=767) | 734 (95.7) | 94.3 - 97.1 | 0.07 |
| $40,000- <$60,000 (N=338) | 326 (96.4) | 94.5 - 98.4 | |
| $60,000 - $100,000 (N=360) | 355 (98.6) | 97.4 - 99.8 | |
| > $100,000 (N=276) | 269 (97.5) | 95.6 - 99.3 | |
| **Mental and psychiatric illnesses**[a] | | | |
| Yes (N=1,983) | 1,924 (97.0) | 96.3 - 97.8 | 0.91 |
| No (N=4,651) | 4,532 (97.4) | 97 - 97.9 | |
| **HIV/AIDS** | | | |
| Yes (N=244) | 221 (90.6) | 86.9 - 94.2 | 0.35 |
| No (N=6,390) | 6,325 (99.0) | 98.7 - 99.2 | |
| **Alcohol use disorder** | | | |
| Yes (N=713) | 674 (94.5) | 92.9 - 96.2 | 0.57 |
| No (N=5,921) | 5,782 (97.7) | 97.3 - 98 | |
| **Injection drug use** | | | |
| Yes (N=809) | 769 (95.1) | 93.6 - 96.5 | 0.62 |
| No (N=5,825) | 5,687 (97.6) | 97.2 - 98 | |

© 2022 Nguyen VH et al. *JAMA Network Open.*

HCV, hepatitis C virus; HCC, hepatocellular carcinoma; CI, confidence interval; GI, gastroenterologist; ID, infectious disease; APP, advanced practice provider; HIV, human immunodeficiency viruses; AIDS, acquired immunodeficiency syndrome, SVR, sustained virologic response

[a]Mental and psychiatric illnesses include post-traumatic stress disorders, anxiety, panic disorders, depression, bipolar disorder, mania, mood disorders, delusion, psychosis, schizoaffective disorder, schizophrenia, dementia

© 2022 Nguyen VH et al. *JAMA Network Open.*
