# Supplementary material for: Characteristics and Treatment Rate of Patients With Hepatitis C Virus Infection in the Direct-Acting Antiviral Era and During the COVID-19 Pandemic in the United States
Source: JAMA Netw Open. 2022 Dec 7;5(12):e2245424. doi: 10.1001/jamanetworkopen.2022.45424 (PMC9856330; doi:10.1001/jamanetworkopen.2022.45424)
Supplement: Supplement 2. — Data Sharing Statement [file jamanetwopen-e2245424-s002.pdf]

Data Sharing Statement

Nguyen. Characteristics and Treatment Rate of Patients With Hepatitis C Virus Infection in the Direct-Acting Antiviral Era and During the COVID-19 Pandemic in the United States. *JAMA Netw Open*. Published December 07, 2022. doi:10.1001/jamanetworkopen.2022.45424

Data
Data available: No

Additional Information
Explanation for why data not available: Individual patient data can only be obtained with permission from Optum. Aggregated data we have produced have been submitted in the manuscript.
